# Genome-wide identification, characterization, and expression analysis of the NAC transcription factor family in orchardgrass (*Dactylis glomerata* L.)

**DOI:** 10.1186/s12864-021-07485-6

**Published:** 2021-03-12

**Authors:** Zhongfu Yang, Gang Nie, Guangyan Feng, Jiating Han, Linkai Huang, Xinquan Zhang

**Affiliations:** College of Grassland Science and Technology, Sichuan Agricultural University, Chengdu, 611130 Sichuan Province China

**Keywords:** Orchardgrass, *NAC* genes, Gene expression, Floral bud development, Stress response, Phylogenetics

## Abstract

**Background:**

Orchardgrass (*Dactylis glomerata* L.) is one of the most important cool-season perennial forage grasses that is widely cultivated in the world and is highly tolerant to stressful conditions. However, little is known about the mechanisms underlying this tolerance. The *NAC (NAM*, *ATAF1/2*, and *CUC2*) transcription factor family is a large plant-specific gene family that actively participates in plant growth, development, and response to abiotic stress. At present, owing to the absence of genomic information, *NAC* genes have not been systematically studied in orchardgrass. The recent release of the complete genome sequence of orchardgrass provided a basic platform for the investigation of DgNAC proteins.

**Results:**

Using the recently released orchardgrass genome database, a total of 108 *NAC* (*DgNAC*) genes were identified in the orchardgrass genome database and named based on their chromosomal location. Phylogenetic analysis showed that the DgNAC proteins were distributed in 14 subgroups based on homology with NAC proteins in *Arabidopsis*, including the orchardgrass-specific subgroup Dg_NAC. Gene structure analysis suggested that the number of exons varied from 1 to 15, and multitudinous *DgNAC* genes contained three exons. Chromosomal mapping analysis found that the *DgNAC* genes were unevenly distributed on seven orchardgrass chromosomes. For the gene expression analysis, the expression levels of *DgNAC* genes in different tissues and floral bud developmental stages were quite different. Quantitative real-time PCR analysis showed distinct expression patterns of 12 *DgNAC* genes in response to different abiotic stresses. The results from the RNA-seq data revealed that orchardgrass-specific *NAC* exhibited expression preference or specificity in diverse abiotic stress responses, and the results indicated that these genes may play an important role in the adaptation of orchardgrass under different environments.

**Conclusions:**

In the current study, a comprehensive and systematic genome-wide analysis of the *NAC* gene family in orchardgrass was first performed. A total of 108 *NAC* genes were identified in orchardgrass, and the expression of *NAC* genes during plant growth and floral bud development and response to various abiotic stresses were investigated. These results will be helpful for further functional characteristic descriptions of *DgNAC* genes and the improvement of orchardgrass in breeding programs.

**Supplementary Information:**

The online version contains supplementary material available at 10.1186/s12864-021-07485-6.

## Background

Transcription factors (TFs) are deemed to govern cellular processes in plants, such as signal transduction, cellular morphogenesis, and resistance to environmental stress [1, 2]. Generally, TFs regulate gene expression by binding to specific cis-acting promoters to activate or inhibit the transcription level of target genes [3, 4]. Among them, NAC is one of the largest and most plant-specific TF families and is named according to three proteins: petunia no apical meristem (NAM), *Arabidopsis thaliana* ATAF1/2 and cup-shaped cotyledon (CUC) [5, 6]. Typical NAC proteins include a highly conserved N-terminal region (NAC domain), which comprises five subdomains (A–E), whereas the C-terminal region contains a transcriptional activation/repression region (TAR or TRR) that is relatively divergent [5, 7, 8]. The subdomains of NAC domains are relevant to DNA binding, dimer formation and localization [8–11]. In addition, compared with subdomains B and E, subdomains A, C, and D are highly conserved [12–15]. The C-terminal regions might also be involved in protein-protein interactions and contribute to their regulation specificities [16].

NAC transcription factors play a critical role in the regulation of plant growth and development. In *Arabidopsis thaliana*, *AtNAC1* and *AtNAC2* are involved in lateral root development by downregulating auxin signals [17], while *NAP is* related to leaf senescence [18] and floral morphogenesis [19]. In addition, *NTL8* controls seed germination by regulating gibberellic acid-mediated salt signaling [20] and regulates trichome formation by activating target genes (*TRY* and *TCL1*) in *Arabidopsis* [21]. In a previous study, it was reported that *ORE1* could positively regulate aging-induced cell death in *Arabidopsis* leaves [22]. The NAC TFs of *ONAC020/023/026* were associated with seed size/weight in rice (*Oryza sativa*) [23]. In cotton (*Gossypium hirsutum*), *GhFSN1* participates in fiber development by activating its downstream secondary cell wall-related genes [24]. The NAC domain transcription factors *NST1* and *NST3* are involved in secondary wall biosynthesis, including the production of xylary and interfascicular fibers and pod shattering [25–27]. In *Medicago truncatula*, loss of *MtNST1* function resulted in reduced lignin content associated with reduced expression of most lignin biosynthetic genes [28].

In addition, *NAC* genes also play an important role in the response to abiotic stresses. In *Arabidopsis thaliana*, *AtNAP* is a negative regulator that represses *AREB1* under salt stress [29]. *ANAC069* recognizes the DNA sequence of C[A/G]CG[T/G], which negatively regulates tolerance to salt and osmotic stress by reducing ROS scavenging capability and proline biosynthesis [30]. In wheat (*Triticum aestivum*), the overexpression of *TaRNAC1* enhances drought tolerance [31]. The overexpression of *TaNAC69* results in enhanced dehydration tolerance and the transcript levels of stress-induced genes in wheat [32]. The overexpression of *TaNAC29* increased salt tolerance by enhancing the antioxidant system to reduce H_2_O_2_ accumulation and membrane damage [33]. Overexpression of *OsNAC6/SNAC2* could also improve the drought, salt and cold tolerance of rice seedlings [34, 35]. In rice, *ONAC022* enhanced drought and salt tolerance by regulating an ABA-mediated pathway [36]. Furthermore, the NAC transcription factor *JUNGBRUNNEN 1* enhances tomato tolerance to drought stress [37]. In *Arabidopsis*, the heteroexpression of the Miscanthus NAC protein *MINAC12* was found to result in activation of ROS scavenging enzymes to improve drought and salt tolerance [38]. A previous study illustrated that NAC genes are related to vernalization and flowering in orchardgrass by transcriptome analysis [39].

Orchardgrass (*Dactylis glomerata* L.) is one of the most important cool-season perennial grasses and is native to Europe and North Africa [40]. Orchardgrass is grown widely across the world due to its high biomass and nutritional quality, good shade, drought and barren tolerance, and high feed quality [41]. In addition, orchardgrass is also an important species in rocky desertification control in southwestern China. Therefore, orchardgrass has great economic and ecological value, and identification of functional genes is required to improve orchardgrass productivity. *NAC* genes have been widely studied in various plant species, such as *Arabidopsis thaliana* [13], *Oryza sativa* [7], *Zea mays* [42], *Glycine max* [43], *Solanum tuberosum* [44], *Pyrus bretschneideri* [45], *Fagopyrum tataricum* [46], and *Panicum miliaceum* [47]. However, the *NAC* gene family in orchardgrass has not been systematically studied. With the completion of *Dactylis glomerata* L. genome sequencing, a systematic analysis of the *NAC* family during orchardgrass is expected to accelerate molecular breeding in orchardgrass [48]. In this study, we identified 108 orchardgrass *NAC* genes and classified them into 14 subgroups, including the orchardgrass-specific subgroup Dg_NAC. Comprehensive and systematic characteristics, including gene structure, conserved motif compositions, chromosomal distribution, gene duplications and phylogenetic characteristics, and homologous relationships were further investigated. In addition, the expression of *DgNAC* genes during plant growth and floral bud development and the response to various abiotic stresses were analyzed. The present results will be useful for illustrating the molecular mechanisms of orchardgrass adaptability under various environmental conditions, further analysis of the functional characteristics of candidate *DgNAC* genes and providing valuable clues for molecular assisted breeding in orchardgrass.

## Results

### Identification of the *DgNAC* genes in orchardgrass

Members of the *NAC* family were identified in the orchardgrass genome using the Hidden Markov Model (HMM) search with the HMM profile (PF02365) of the NAM domain. A total of 108 candidate gene models were matched across the whole genome and designated *DgNAC001* to *DgNAC108* based on their order on the chromosomes (Additional file 1). The basic information of 108 DgNAC genes was analyzed in this study, including the CDS length, protein sequence length, relative molecular weight (MW), and isoelectric point (pI) (Additional file 1). The protein sequence length of all DgNAC proteins ranged from 134 (*DgNAC031*) to 938 (*DgNAC094*) amino acids. The MW of the proteins varied from 14.70 to 181.91 kDa. The pI ranged from 4.28 (*DgNAC042*) to 10.25 (*DgNAC012*), with an average of 6.79, suggesting that most DgNAC proteins were weakly acidic.

### Phylogenetic analyses and classification of *DgNAC* genes

**Fig. 1:**
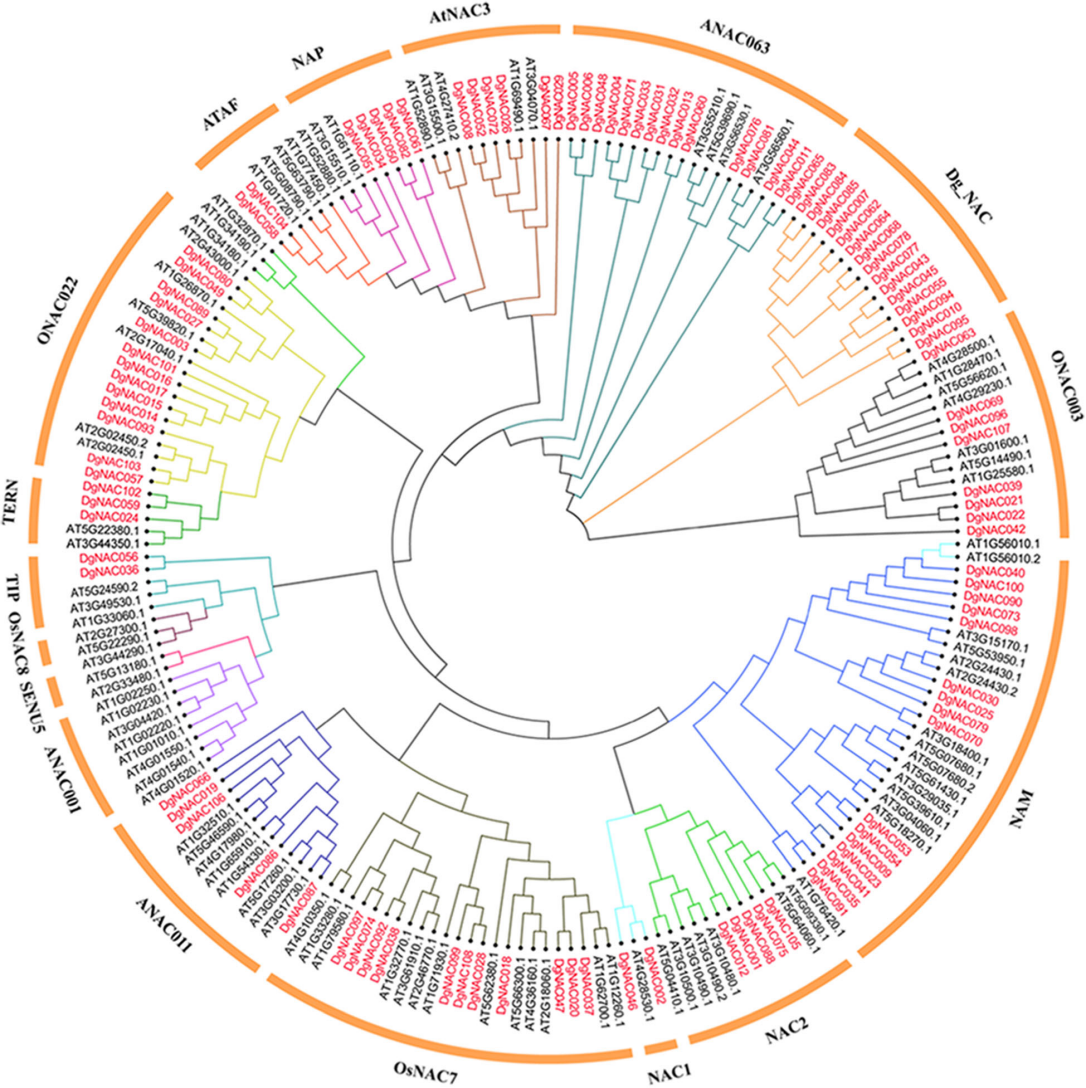
Unrooted phylogenetic tree representing relationships among the NAC proteins of *Dactylis glomerata* and *Arabidopsis thaliana*. The tree divided the DgNAC proteins into 14 subgroups represented by different colored clusters within the tree. A phylogenetic tree was constructed from the NAC protein sequence of *Dactylis glomerata* and *Arabidopsis thaliana*. The phylogenetic tree was derived using the neighbor-joining (NJ) method in Geneious 2020. The parameters used included a Blosum62 cost matrix, the Jukes-Cantor model, global alignment and bootstrap value of 1000

To explore the evolutionary relationship of the *NAC* gene family in orchardgrass, an unrooted phylogenetic tree was constructed by using the amino acid sequences of DgNACs and AtNACs (Fig. 1). The results showed that 108 *DgNAC* genes could be divided into 14 subgroups, including an orchardgrass-specific subgroup named Dg_NAC. As shown in Fig. 1, the NAC proteins of orchardgrass were distributed in the ONAC003, ANAC063, AtNAC3, NAP, ATAF, ONAC022, TERN, TIP, ANAC011, OsNAC7, NAC1, NAC2, and NAM subgroups and orchardgrass-specific subgroup DgNAC. However, in orchardgrass, no NAC members were identified from the OsNAC8, SENU5, and ANAC001 subgroups. Among the 108 DgNAC proteins, only one DgNAC protein belonged to NAC1, the subgroups NAP, ANAC011 and NAC2 contained five DgNAC proteins each, and the orchardgrass-specific subgroup Dg_NAC included 15 DgNAC proteins, whereas the NAM subgroup contained the most DgNAC proteins (16).

### Gene structure and protein motif analysis of *DgNAC* genes

**Fig. 2:**
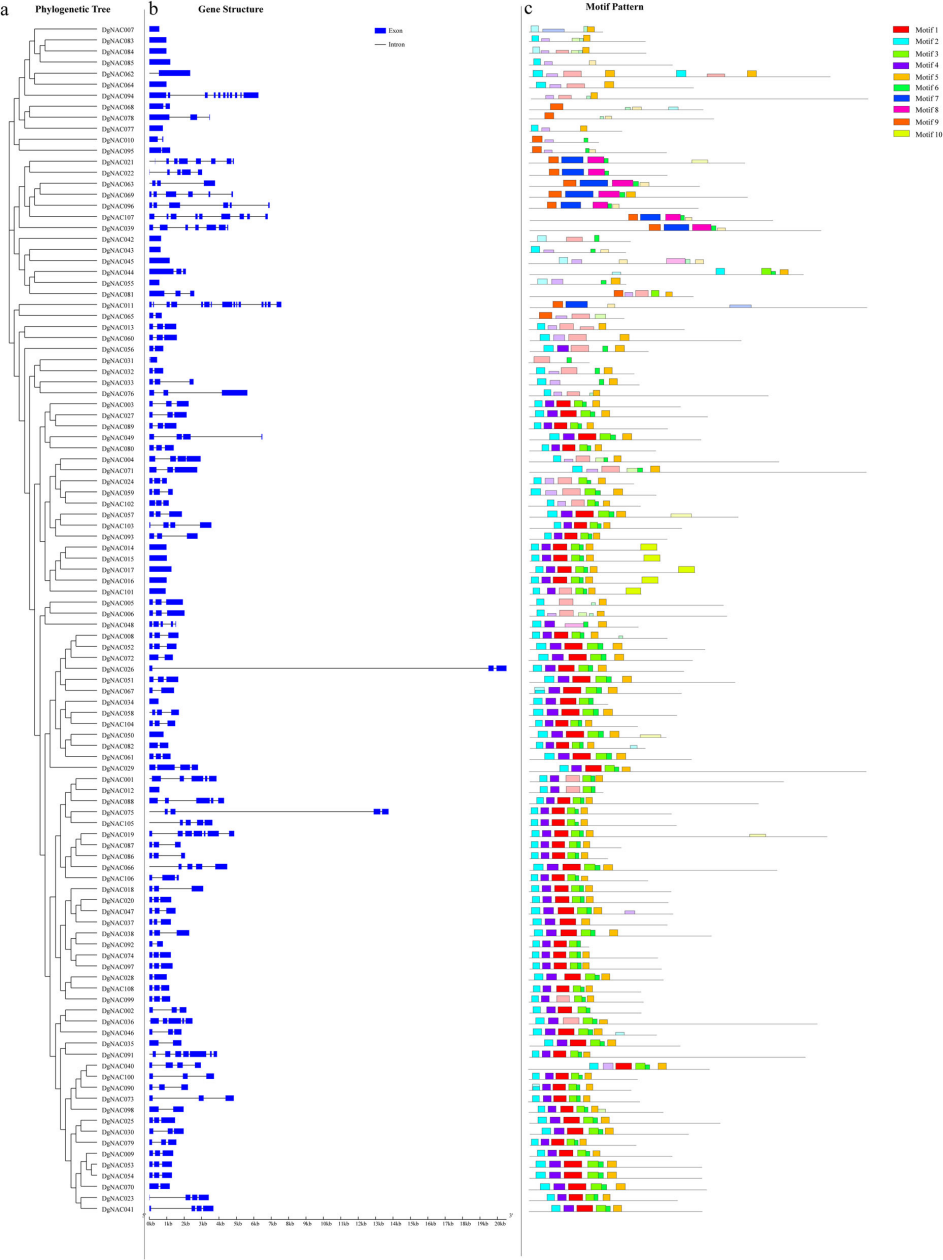
Phylogenetic relationships, gene structure and architecture of conserved protein motifs in *NAC* genes from *Dactylis glomerata*. **a** The phylogenetic tree was constructed based on the full-length sequences of *Dactylis glomerata* NAC proteins using Geneious 2020 software. **b** Exon-intron structure of *Dactylis glomerata NAC* genes. Blue boxes indicate exons; black lines indicate introns. **c** The motif composition of *Dactylis glomerata* NAC proteins. The motifs, numbered 1–10, are displayed in different colored boxes. The sequence information for each motif is provided in Additional file 2

To obtain more insights into the evolution of the *NAC* family in orchardgrass, the structural features of all the identified *DgNAC* genes were analyzed. As shown in Fig. 2b, among the *DgNAC* genes, 17 (approximately 15.74%) were intronless, 20 (12.96%) had one exon, nearly half (50, 46.30%) had three exons, and only 2 genes (*DgNAC011* and *DgNAC094*, with 15 and 11 exons, respectively) had more than ten exons. Among the 15 orchardgrass-specific NAC genes, more than half (10, 66.67%) had only one exon.

To reveal the protein structural diversification of DgNAC proteins, 10 conserved motifs were identified by MEME (Fig. 2c). The amino acid sequences of each motif are listed in Additional file 2. The lengths of these conserved motifs varied from 10 to 55 amino acids. Motifs-1, − 2, − 3, and − 5 were the most conserved parts (Fig. 2c). The orchardgrass-specific NACs DgNAC068 and DgNAC078 contain one type of motif, whereas DgNAC035 contains the highest number of motifs (8 types). The motifs of DgNAC members within the same subgroups display similar patterns, indicating that the same subgroup of genes have similar functions. However, the specific biological function of most of these motifs is unclassified and remains to be further investigated.

### Chromosomal locations and synteny analysis of *DgNAC* genes

**Fig. 3:**
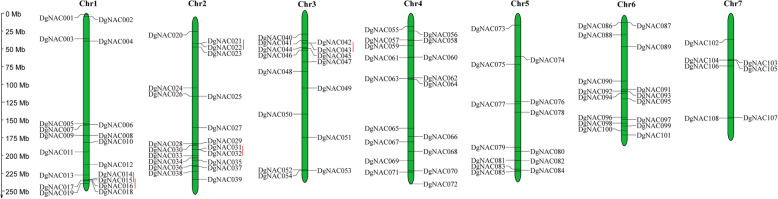
Distribution of *DgNAC* genes among 7 chromosomes. Tandem duplications were connected by thick red line. Vertical bars represent the chromosomes of *Dactylis glomerata*. The chromosome number is to the top of each chromosome. The scale on the left represents chromosome length

To clarify the distribution of *DgNAC* genes on 7 chromosomes of orchardgrass, the MG2C program was used to map *DgNAC* genes on the chromosome (Fig. 3). A total of 108 *DgNACs* were randomly designated onto 7 chromosomes. Chromosome 2 had the highest number of *DgNAC* genes (20, 18.5%), and chromosome 7 harbored the lowest number (7, 6.5%). The orchardgrass-specific NAC genes are distributed on chromosomes 1, 3, 4, 5 and 6, and one-third of them are on chromosome 5. The duplication events of *DgNAC* genes were also examined in this study. The results showed that only 5 pairs of genes of tandem duplicates in the *DgNAC* gene family were identified, including *DgNAC14/15*, *DgNAC15/16*, *DgNAC21/22*, *DgNAC31/32*, and *DgNAC42/43*, and they were linked with the red line, (Fig. 3). The tandem duplicated genes were present on chromosomes 1, 2, and 3, and only one pair of genes was common on chromosome 3.

**Fig. 4:**
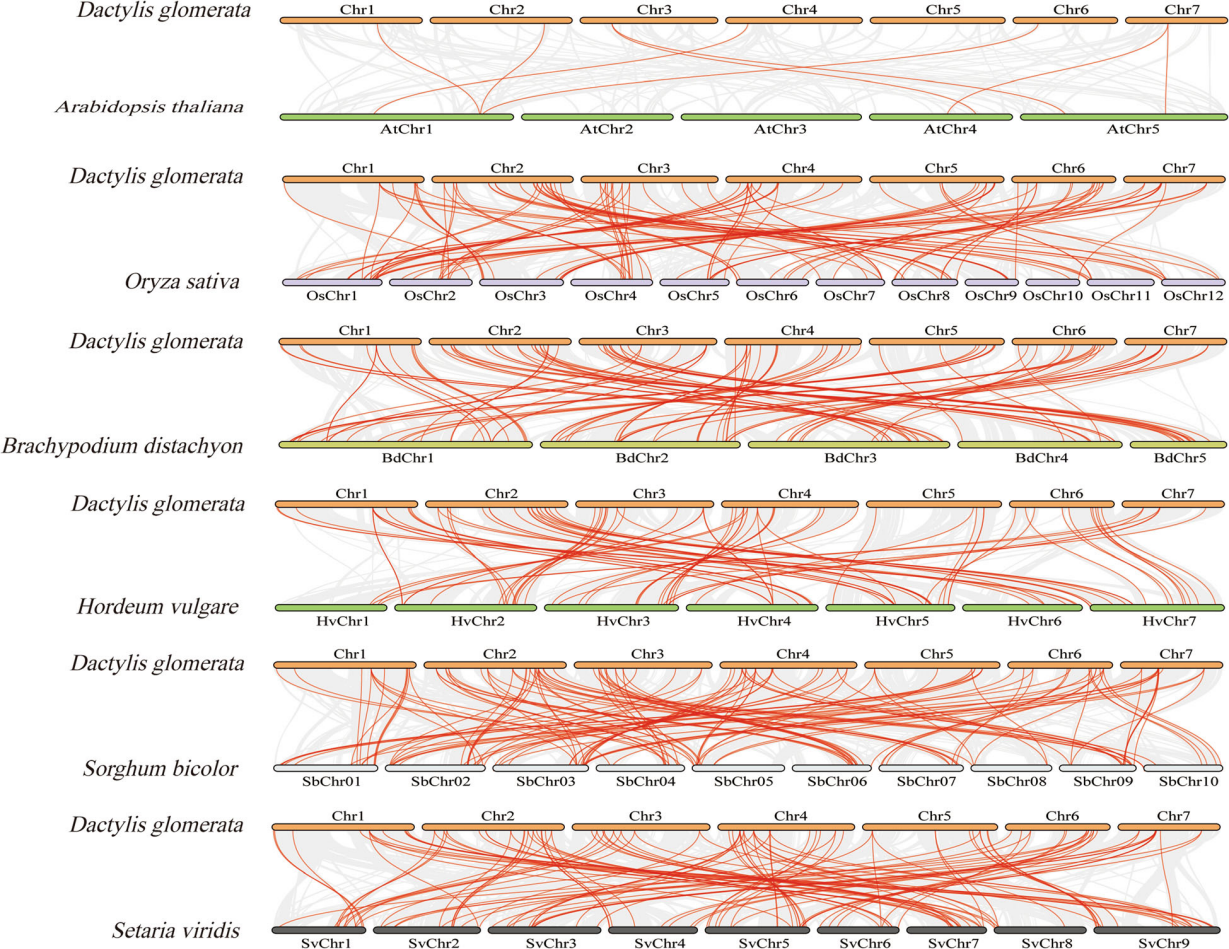
Synteny analysis of *NAC* genes between *Dactylis glomerata* and six representative plant species. Gray lines in the background indicate the collinear blocks within the *Dactylis glomerata* and other plant genomes, whereas the red lines highlight the syntenic *NAC* gene pairs

To further explore the evolutionary relationship of the *NAC* gene family in orchardgrass, five comparative syntenic maps were constructed, which consisted of a dicotyledonous plant (*Arabidopsis thaliana*) and five monocotyledonous plants (*Oryza sativa*, *Brachypodium distachyon*, *Hordeum vulgare*, *Sorghum bicolor* and *Setaria viridis*) (Fig. 4). Seventy-seven *DgNAC* genes showed a syntenic relationship with *Brachypodium distachyon*, *Setaria viridis* (69), *Oryza sativa* (69), *Hordeum vulgare* (68), *Sorghum bicolor* (64) and *Arabidopsis thaliana* (6) (Additional file 3). The number of homologous pairs between the other six species (*Sorghum bicolor*, *Setaria viridis*, *Oryza sativa*, *Brachypodium distachyon*, *Hordeum vulgare* and *Arabidopsis thaliana*) was 145, 114, 107, 98, 84 and 8, respectively.

### Expression profiling of *DgNAC* genes in different tissues based on RNA-seq data

**Fig. 5:**
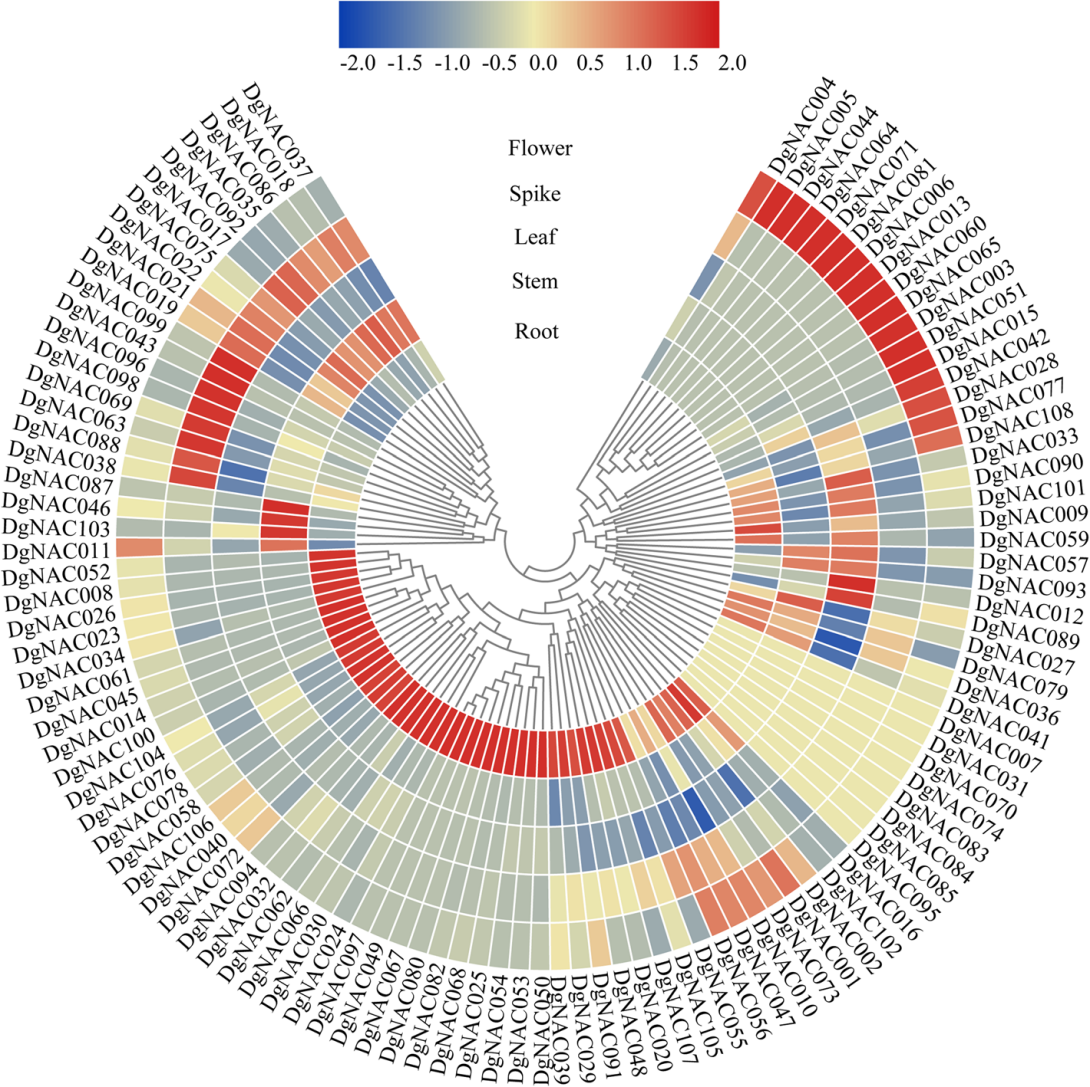
Expression patterns of *DgNAC* genes in different tissues

To better understand the function of *DgNAC* genes in orchardgrass, the transcript levels of *DgNAC* genes in different tissues were examined via the transcriptome data of different orchardgrass tissues derived from the orchardgrass genome database (Fig. 5, Additional file 5). Among the 108 *DgNAC* genes, eight *DgNACs* (*DgNAC007/031/070/074/083/084/085/095*) were not expressed in all detected samples, which may be pseudogenes or have special spatiotemporal expression patterns. Forty-two genes in roots, 3 genes in stems, 3 genes in leaves, 8 genes in spikes, and 17 genes in flowers presented high transcript abundances and may play a critical role in tissue development.

### Expression profiling of *DgNAC* genes in different floral bud development stages with RNA-seq data

**Fig. 6:**
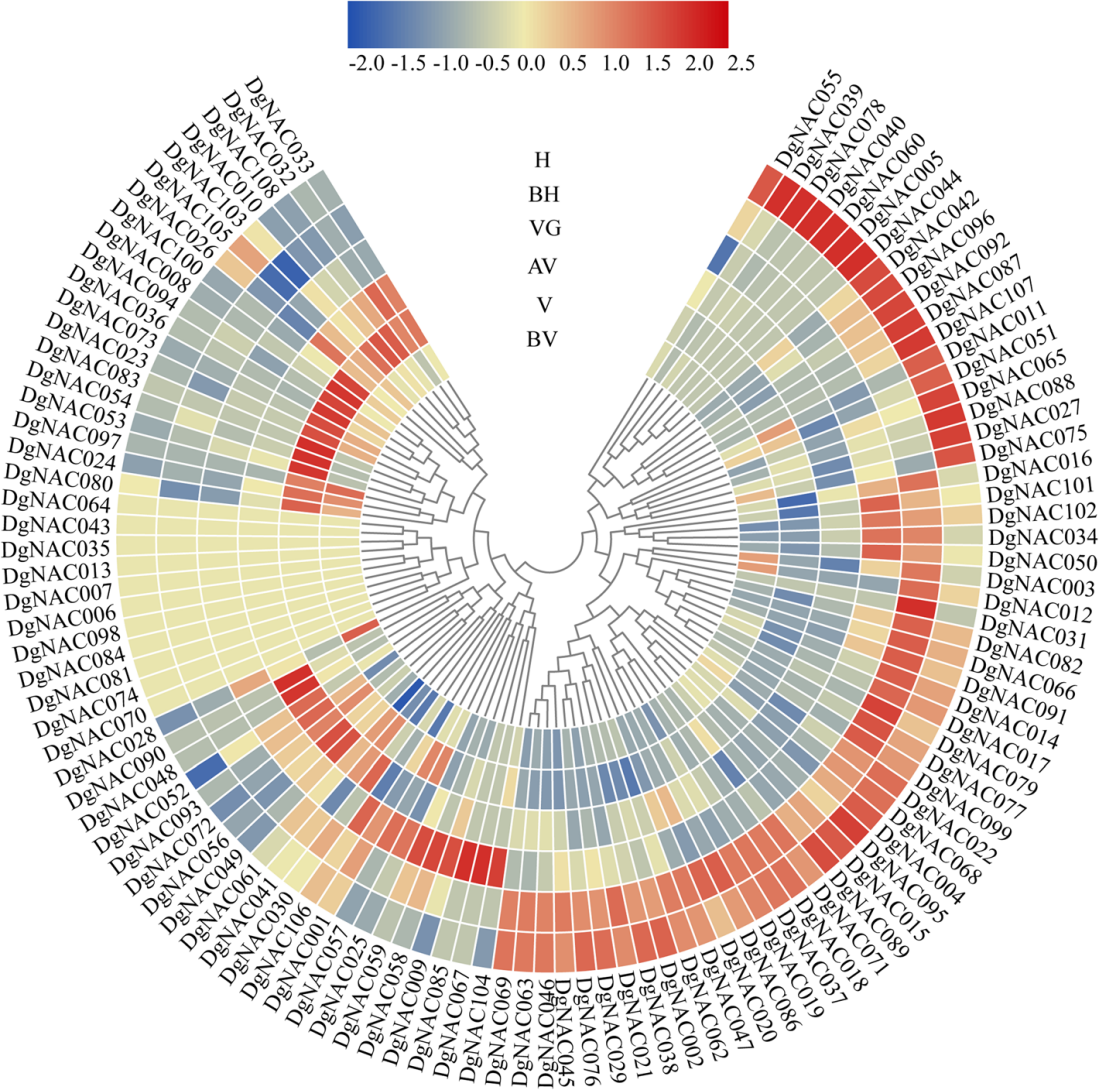
Expression patterns of *DgNAC* genes in different floral bud development stages. BV, before vernalization; V, vernalization; AV, after vernalization; VG, vegetative growth; BH, before heading, and H, heading stage

To further analyze the role of *NAC* genes in the regulation of orchardgrass flowering, we used RNA-seq data to analyze the transcript levels of all 108 *DgNAC* genes in different floral bud development stages. The *DgNAC* genes exhibited different expression profiles with floral bud development. Several *DgNAC* genes presented similar expression patterns from the before vernalization (BV) stage to the heading (H) stage, such as *DgNAC087* and *DgNAC107,* with gradually increased expression levels (Fig. 6, Additional file 6). Some genes showed preferential expression during the floral bud development of orchardgrass. Among them, eleven genes in the vernalization stage, four genes (*DgNAC048/049/056/090*) in the after vernalization stage, and twenty genes in the heading stage showed high transcript abundances. These *DgNAC* genes may play a critical role in the different floral development stages. In addition, the special temporal expression patterns of *DgNAC* genes may be related to changes in environmental conditions. For example, *DgNAC* genes respond to low temperatures in vernalization and long days in the heading stage.

### Expression patterns of *DgNAC* genes in response to different abiotic stress

**Fig. 7:**
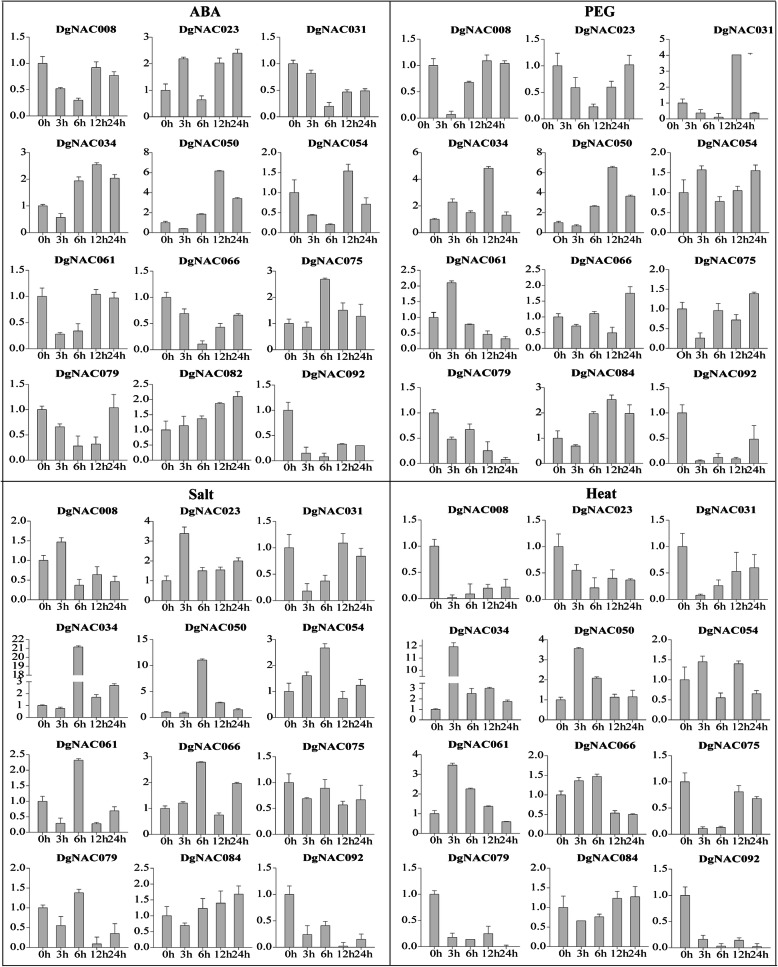
Expression profiles of 12 selected *DgNAC* genes in response to various abiotic stress treatments. Data were normalized to *GAPDH* gene and vertical bars indicate standard deviation

Gene expression patterns can provide crucial information for determining gene function. To investigate the role of *NAC* genes in orchardgrass under various abiotic stresses, 12 *DgNAC* members were selected for quantitative expression analysis in response to ABA, PEG, heat, and salt treatment durations (Fig. 7). Some *DgNAC* genes were induced/repressed by multiple treatments, such as *DgNAC092* was inhibited by ABA, PEG, heat, and salt treatments, and *DgNAC023* was induced by salt and ABA treatment after 3 h. In contrast, multiple *DgNAC* genes can be induced simultaneously by the same treatment. For instance, four *DgNAC* genes (*DgNAC034/050/075/082*) were induced by ABA treatment, and six genes (*DgNAC034/050/054/061/066/084*) were induced by salt treatment. Interestingly, the expression level of *DgNAC034* was higher than that of other selected genes under salt and heat treatment. The expression levels of many *DgNAC* genes, such as *DgNAC008*, *DgNAC023*, *DgNAC079* and *DgNAC092,* were reduced by heat treatment. Furthermore, some genes showed opposing expression patterns under different treatments; for example, *DgNAC023* was induced by ABA and salt but repressed by heat treatment.

**Fig. 8:**
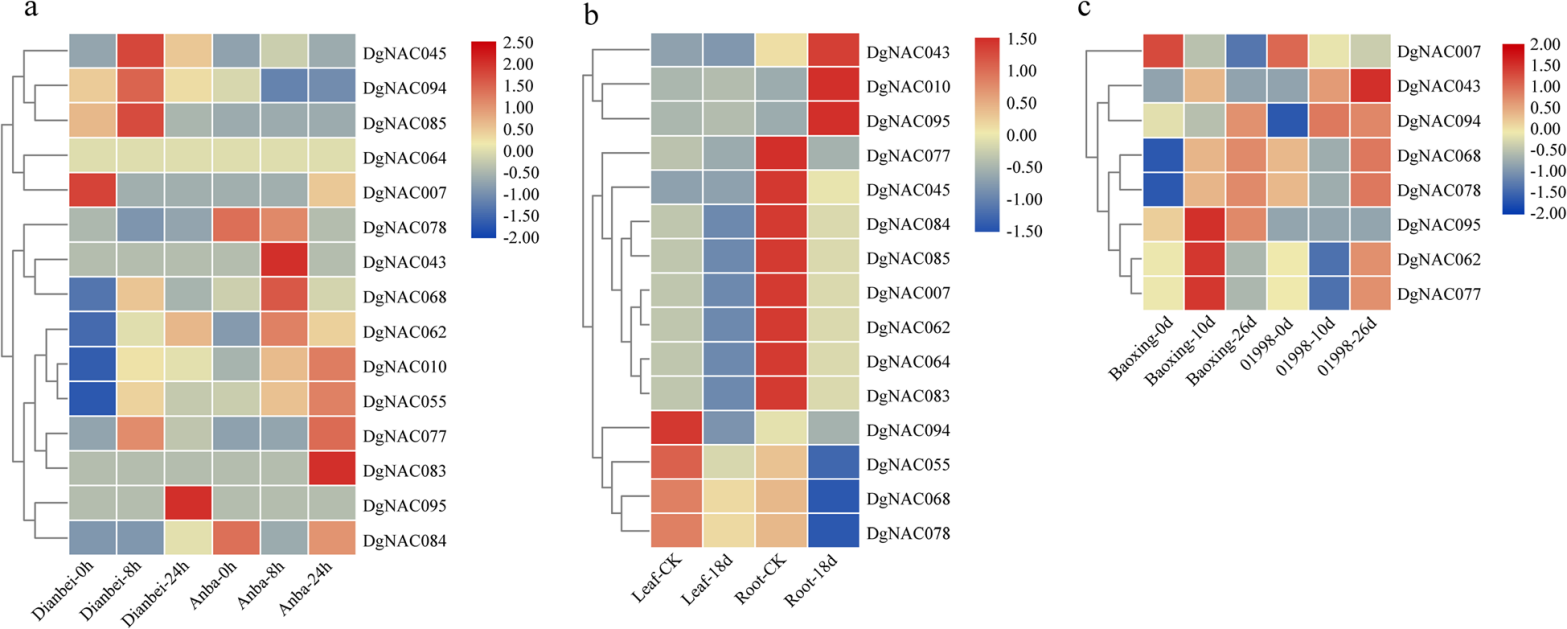
Expression profiles of orchardgrass-specific *NAC* genes in response to submergence stress, drought stress, and heat stress. **a** The heat map of *DgNAC* genes in submergence treatment for 0, 8, and 24 h, ‘Dianbei’ is submergence tolerant and ‘Anba’ is submergence sensitive. **b** the heat map of *DgNAC* genes in leaf and root of highly drought-resistant variety ‘Baoxing’ under drought treatment for 0 and 18d. **c** the heat map of part of orchardgrass-specific *NAC* genes under heat treatment for 0, 10, and 26d, ‘Baoxing’ is heat resistant, ‘01998’ is heat sensitive

To understand the potential function of orchardgrass-specific *NAC* genes in resisting environmental stress, we also analyzed the transcriptional levels of *DgNAC* genes from the Dg_NAC subgroup. The results showed that Dg_NACs are differentially expressed under submergence and heat tolerance (Fig. 8). In the submergence-tolerant cultivar ‘Dianbei’, *DgNAC045*, *DgNAC094* and *DgNAC085* were significantly upregulated after submergence treatment for 8 h (Fig. 8a). For drought stress treatment (18 d), the expression of *DgNAC043*, *DgNAC010*, and *DgNAC095* was significantly upregulated in the roots of the tolerant variety ‘Baoxing’ (Fig. 8b). Under heat conditions, *DgNAC062* and *DgNAC077* were significantly upregulated in the heat-resistant variety ‘Baoxing’, while these two genes were downregulated in the heat-susceptible variety ‘01998’ (Fig. 8c).

## Discussion

### *DgNAC* gene identification and evolutionary analysis in orchardgrass

The *NAC* gene family is an important transcription factor in plants that plays roles in the regulation of growth, development, and stress responses [49–51]. Genome-wide identification of *NAC* genes has been studied in many plant species, while little is known about this gene family in the high-quality forge *D. glomerata*. In this study, a total of 108 *NAC* genes were identified based on the *D. glomerata* genome database [48], which was higher than the 104 *NAC* genes identified in *Capsicum annuum* [52], 82 *NAC* genes identified in *Cucumis melo* [53], 80 *NAC* genes identified in *Fagopyrum tataricum* [46], and 96 *NAC* genes identified in *Manihot esculenta* [54] but lower than the 115 *NAC* genes identified in *Arabidopsis thaliana* [13], 151 *NAC* genes identified in *Oryza sativa* [55], 152 *NAC* genes identified in *Zea mays* [42], 152 *NAC* genes identified in *Glycine max* [43], 110 *NAC* genes identified in *Solanum tuberosum* [44], and 204 *NAC* genes identified in Chinese cabbage [56]. Evidence from physical and chemical parameters and gene structure and protein motifs confirms that genes originating from progenitors can gradually evolve and expand. Duplication events are important in the rapid expansion and evolution of gene families, and the size difference might be due to the more duplication events that occurred in other species after differentiation from their earliest ancestors. For example, the orchardgrass genome experienced one genome duplication event [17], while the *Arabidopsis* genome went through five such events [57]. A collinearity analysis demonstrated that there were 5 pairs of tandem replications without segmental duplication events (Fig. 3). Tandem replication of *NAC* genes has been observed in many species, such as *Arabidopsis thaliana*, *Oryza sativa*, *Solanum tuberosum*, and *Panicum virgatum*. However, the duplication event of orchardgrass increases the genome size rather than increasing many *NAC* gene members, which may be related to the expansion of long terminal repeat retrotransposons (LTR-RTs) [48].

The unrooted tree was constructed using *NAC* protein sequences from orchardgrass and *Arabidopsis* to explain the phylogenetic relationship. According to the sequence homology with *Arabidopsis*, all 108 *DgNAC* genes were divided into 13 subgroups [13]. The results were inconsistent with other species, such as *Fagopyrum tataricum* (15 subgroups) [46], *Capsicum annuum* (14 subgroups) [52] and *Capsicum annuum* (12 subgroups) [47], suggesting that NAC proteins exhibit diversity in various species. The results of conserved motif analysis of orchardgrass NAC proteins further confirm the classification of the *DgNAC* family. Only 8 pairs of homologous genes were found in orchardgrass and *Arabidopsis* by collinearity analysis, whereas more homologous gene pairs were identified in the five monocotyledons, including those of *Oryza sativa*, *Brachypodium distachyon,* and *Hordeum vulgare* (Fig. 4). The results indicated that the NAC genes are more homologous and conserved in monocotyledons.

### Expression patterns and functional prediction of the *DgNAC* genes

Generally, the expression level of a gene determines its function, while the functions of genes are related to their expression patterns [58]. Transcription factors usually play a key role in controlling the expression of tissue-specific genes [59–61]. In this study, the tissue-specific expression pattern showed that more than 40 *DgNAC* genes exhibited higher expression in roots than other detected orchardgrass tissues, such as *DgNAC008/052/026/023/034/061/045*. Similar results were also found in other plants, such as *Fagopyrum tataricum* [46], *Panicum miliaceum* [47] and *Triticum aestivum* [62]. *DgNAC046, DgNAC087,* and *DgNAC103* exhibited higher expression than the other genes in the stems of orchardgrass, and they may play an important role in stem development. In addition, previous studies have demonstrated that the development of tissues could be promoted by overexpression of tissue-specifically expressed *NAC* genes, such as *NAC15* from poplar, which enhanced wood formation [63], and the *NAC* domain transcription factor *PdWND3A* affected lignin biosynthesis and composition in populous [64]. In general, genes in one branch of the phylogenetic tree often have the same function and similar expression profiles. Although *DgNAC021* and *DgNAC022* are duplicated genes within the same subgroup, the expression pattern of *DgNAC021* was different from that of *DgNAC022*, which might be caused by variation in gene regulation after duplication events, and the differential expression patterns of duplicated *DgNAC* genes indicated that they might have experienced functionalization during the evolutionary process [65, 66]. The NAM subgroups may regulate cell division and leaf development [67–72], and the gene *DgNAC090* is most highly expressed in the leaf followed by the root, indicating that *DgNAC090* may function in leaf development and cell division through expression in both the leaf and root (Fig. 5). These results demonstrated that *DgNAC* genes are widely involved in the tissue development of orchardgrass.

Orchardgrass is a high-quality perennial forage grass, and flowering time is a critical factor affecting forage quality and utilization. In the current study, the potential role of *NAC* genes in the regulation of orchardgrass flowering time was investigated by using transcriptome data. The *DgNAC* genes were most highly expressed in different floral bud development stages (Fig. 6). Among them, *DgNAC033* had a special expression pattern during the vernalization and after vernalization stages in orchardgrass, suggesting that it has an important function in the induction of flower primordia. A previous study indicated that the *CUC1* gene regulates shoot apical meristem formation in *Arabidopsis* [72]. After vernalization of orchardgrass, three *DgNAC* genes (*DgNAC034/050/082*) showed high expression in vegetable growth and before the heading stage, indicating that these genes may play an important role in young inflorescence development and regulation of flowering time. The overexpression of the *BnNAC485* gene in *Brassica napus* alters flowering time [73]. In *Arabidopsis*, *NAC050* and *NAC052* are involved in transcriptional repression and flowering time control by associating with the histone demethylase *JMJ14* [74]. Overall, the expression of *DgNAC* genes varies in different floral bud development stages, which potentially regulates orchardgrass flowering time.

Orchardgrass is a widely adapted perennial forage grown on all continents. Orchardgrass is more tolerant of shade, drought, and heat than other cool-season perennial grasses. In plants, most of the *NAC* genes involved in the response to abiotic stress, such as drought, salinity, and heat, have been studied. However, there are few reports of *NAC* genes involved in the abiotic stress response in orchardgrass. Therefore, one of the goals of this study was to obtain more insights into the expression patterns and putative functions of *DgNAC* genes in response to various abiotic stresses. The expression levels of 12 *DgNAC* genes under four stress treatments (ABA, PEG, salt, and heat) were calculated (Fig. 7). All 12 *DgNAC* genes were induced by these treatments; in particular, *DgNAC034* and *DgNAC050* were significantly upregulated after PEG treatment for 12 h, salt treatment for 6 h, and heat treatment for 3 h, and *DgNAC092* was repressed by all treatments. The expression pattern of orchardgrass-specific *NAC* genes under submergence, drought, and heat stress showed that *NAC* may play an important role in orchardgrass adaptation and resistance to various environmental stresses. These results provide new insight into how the accumulation of *DgNAC* effectively reduces abiotic stress damage.

## Conclusions

In the current study, a comprehensive and systematic genome-wide analysis of the NAC gene family in orchardgrass was first performed. A total of 108 *DgNAC* genes were identified and classified into 14 subgroups, including the orchardgrass-specific subgroup Dg_NAC. Comprehensive and systematic characteristics, including gene structure, conserved motif compositions, chromosomal distribution, gene duplications and phylogenetic characteristics, and homologous relationships were further investigated. In addition, the expression of *DgNAC* genes in various tissues, developmental stages of floral bud development, and responses to various abiotic stresses implied that *DgNAC* may participate in the development and stress tolerance of orchardgrass. These results are useful for revealing the adaptability of orchardgrass under various environmental stresses. This comprehensive analysis of the *NAC* gene in orchardgrass is a valuable resource for further studying the functional characteristics of *DgNAC* genes and cultivating high-quality orchardgrass varieties.

## Methods

### Identification of *NAC* genes in orchardgrass

The orchardgrass genome resources were downloaded from the orchardgrass genomics database (http://orchardgrassgenome.sicau.edu.cn/) [48]. For the identification of NAC proteins, the hidden Markov model (HMM) file of the NAM domain (PF02365) was downloaded from the Pfam database (http://pfam.sanger.ac.uk/) as the query [75]. HMMER 3.0 was used to scan the annotated protein with the NAM HMM file. The proteins acquired through the NAM HMM were aligned by ClustalW (E-value <1e-^20^) and used to rebuild an orchardgrass-specific NAM HMM file using hmmbuild in HMMER 3.0. The orchardgrass-specific NAM HMM was used to identify the NAC proteins from orchardgrass genome annotations, and the cutoff value was set to 0.01 [76]. The NAM conserved domain of all candidate genes was further confirmed by the Conserved Domains Database (CDD, http://www.ncbi.nlm.nih.gov/cdd/) and PFAM program [75]. Finally, the physical and chemical parameters of the DgNAC proteins were predicted by ProtParam (http://web.expasy.org/protparam/), including the CDS (coding sequence) length, molecular weights (MW), and isoelectric points (PI).

### Phylogenetic analysis and classification of the *DgNAC* gene family

The NAC protein sequences of *Arabidopsis* were downloaded from the *Arabidopsis* genome TAIR 11 (https://www.arabidopsis.org/) [77]. All the identified *DgNAC* genes were assigned into different groups based on the classification of *AtNACs* [13]. Geneious 2020 was used to construct neighbor-joining (NJ) trees with the following parameters: Blosum62 cost matrix, Jukes-Cantor model, global alignment and bootstrap value of 1000.

### Gene structure and motif analysis

The exon-intron display was constructed according to the Gene Structure Display Server (GSDS, http://gsds.gao-lab.org/) program [78] according to the available CDS and genomic information of the *DgNACs*. The Multiple Expectation Maximization for Motif Elicitation (MEME, http://meme-suite.org/tools/meme) program [79] was used to identify the conserved motifs in DgNAC protein sequences with parameters that maximum 10 motifs and range of motif width 6 to 200.

### Chromosomal mapping and gene duplication analysis

The chromosomal positions of the *DgNAC* genes were acquired from the orchardgrass genome annotations. The chromosomal map of *DgNAC* genes was drafted by MapGene2Chrome (MG2C, http://mg2c.iask.in/mg2c_v2.0/). *DgNAC* gene duplication was examined by using MCScanX software with default parameters. The Dual Synteny Plotter of TBtools (https://github.com/CJ-Chen/TBtools) [80] was used to analyze the homology of the *NAC* gene between orchardgrass and the other plants (including *Arabidopsis thaliana*, *Oryza sativa*, *Brachypodium distachyon*, *Hordeum vulgare*, *Sorghum bicolor* and *Setaria viridis*).

### Plant material, growth condition and stress treatments

The *Dactylis glomerata* cv. DONATA (Registered No. 398) seeds were provided by DLF (Beijing, China). The seeds were sown in pots (18.5 cm length, 13.5 cm width, and 5 cm deep) filled with sterilized quartz and ddH_2_O in growth chambers. The parameters of the growth chamber were set as a 22 °C 14 h photoperiod and a 20 °C 10 h dark period. After 1 week of germination, seedlings were irrigated with Hoagland’s solution for another 60 days. Then, the seedlings were separately subjected to various stress treatments, including drought, ABA, salt, and heat. For salt, ABA, and drought treatments, the plants were subjected to 250 mmol NaCl, 100 μmol ABA, and 20% PEG 6000 (W/V) Hoagland’s solution, respectively. For heat treatment, the plants were exposed to high temperature at 40 °C/35 °C (day/night). Several *DgNAC* genes were selected to analyze the expression profile under various stresses by qRT-PCR analysis. The samples were collected at 0, 3, 6, 12 and 24 h after treatments. All materials harvested from each treatment were immediately frozen in liquid nitrogen and stored at − 80 °C before RNA isolation. All experiments were conducted three times with three biological replicates for qRT-PCR analysis.

### RNA isolation, cDNA synthesis, and qRT-PCR

The Hipure HP plant RNA mini kit (Magen, R4165–02) was used to extract total RNA. DNA-free RNA was used for the synthesis of cDNA by using ReverTra Ace® qPCR RT Master Mix (TOYOBO, FSQ-301) according to the manufacturer’s recommendations. qRT-PCR was performed with a Bio-Rad CFX96 instrument using SYBR® green real-time PCR master Mix (TOYOBO, QPK-201). Primers used for qPCR were designed with primer 6.0, and glyceraldehyde 3-phosphate dehydrogenase (*GAPDH*) was selected as the reference gene (Additional file 4) [81]. The detailed methods of reaction and relative quantitative calculations have been described in a previous study [39]. The transcriptome data of various orchardgrass tissues were obtained from the orchardgrass genome database (Additional file 5) [48], and the transcriptome data of vernalization and floral bud development of orchardgrass were obtained from Feng et al. (Additional file 6) [39]. The RNA-seq data of orchardgrass-specific *NAC* genes (Additional file 7) under submergence, drought and heat stress were obtained from Zeng et al. [82], Ji et al. [83], and Huang et al. [84], respectively.

## Supplementary Information

**Additional file 1 **List of the 108 *DgNAC* genes identified in orchardgrass.

**Additional file 2.** Analysis and distribution of conserved motifs in orchardgrass NAC proteins.

**Additional file 3.** One-to-one orthologous relationships between orchardgrass and other plants.

**Additional file 4.** Sequences of the primers used in this study.

**Additional file 5 **Expression (FPKM) of *DgNAC* genes in different tissues.

**Additional file 6 **Expression (FPKM) of *DgNAC* genes in different floral bud development stages.

**Additional file 7 **RNA-seq data of the orchardgrass-specific *NAC* genes that were used in this study.

## Data Availability

All data generated or analyzed during this study are included in this article and its additional files. The orchardgrass genome resources were downloaded from http://orchardgrassgenome.sicau.edu.cn/ [48]; the genome data used for comparative syntenic analysis were obtained from open database, the genome data of *Arabidopsis thaliana* was downloaded from TAIR (https://www.arabidopsis.org/download/index-auto.jsp?dir=%2Fdownload_files%2FSequences%2FAraport11_blastsets) [77], the genome data of *Oryza sativa* was downloaded from Rice Genome Annotation Project (http://rice.plantbiology.msu.edu/pub/data/Eukaryotic_Projects/o_sativa/annotation_dbs/pseudomolecules/version_7.0/all.dir/) [85], the genome data of *Hordeum vulgare* (variety Barke) was downloaded from https://webblast.ipk-gatersleben.de/downloads/barley_pangenome/Barke/ [86], the genome data of *Brachypodium distachyon* (v3.1, https://genome.jgi.doe.gov/portal/pages/dynamicOrganismDownload. jsf?organism = Bdistachyon*)*, *Sorghum bicolor* (v3.1.1, https://genome.jgi.doe.gov/portal/pages/dynamic OrganismDownload.jsf?organism = Sbicolor) and *Setaria viridis* (v2.1, https://genome.jgi.doe.gov/portal/ pages/dynamicOrganismDownload.jsf?organism = Sviridis) were downloaded; the transcriptome data of DgNACs in different tissues also be obtained on the orchardgrass genome website (http://orchardgrassgenome.sicau.edu.cn/) [48], the raw RNA-seq reads of vernalization and floral bud development [39], submergence [82], drought [83] and heat [84] stress of orchardgrass were obtained from NCBI database (accession SRR5341102, PRJNA565626 and PRJNA554779, SRP158919, SRP049315, respectively).

## Abbreviations

ABA: Abscisic acid
CDS: Coding sequence
CUC: Cup-shaped cotyledon
HMM: Hidden Markov model
MW: Molecular weight
NAM: No apical meristem
TFs: Transcription factors
PEG: Polyethylene glycol

## References

[1] Riaño-Pachón DM, Ruzicic S, Dreyer I, Mueller-Roeber B (2007). PlnTFDB: an integrative plant transcription factor database. BMC Bioinformatics.

[2] Zhang H, Jin J, Tang L, Zhao Y, Gu X, Gao G, Luo J (2011). PlantTFDB 2.0: update and improvement of the comprehensive plant transcription factor database. Nucleic Acid Res.

[3] Riechmann JL, Heard J, Martin G, Reuber L, Jiang C-Z, Keddie J, Adam L, Pineda O, Ratcliffe O, Samaha R (2000). Arabidopsis transcription factors: genome-wide comparative analysis among eukaryotes. Science.

[4] Wray GA, Hahn MW, Abouheif E, Balhoff JP, Pizer M, Rockman MV, Romano LA (2003). The evolution of transcriptional regulation in eukaryotes. Mol Biol Evol.

[5] Aida M, Ishida T, Fukaki H, Fujisawa H, Tasaka M (1997). Genes involved in organ separation in Arabidopsis: an analysis of the cup-shaped cotyledon mutant. Plant Cell.

[6] Souer E, van Houwelingen A, Kloos D, Mol J, Koes R (1996). The no apical meristem gene of Petunia is required for pattern formation in embryos and flowers and is expressed at meristem and primordia boundaries. Cell.

[7] Kikuchi K, Ueguchi-Tanaka M, Yoshida K, Nagato Y, Matsusoka M, Hirano H-Y (2000). Molecular analysis of the NAC gene family in rice. Mol Gen Genet MGG.

[8] Puranik S, Sahu PP, Srivastava PS, Prasad M (2012). NAC proteins: regulation and role in stress tolerance. Trends Plant Sci.

[9] Jensen MK, Kjaersgaard T, Nielsen MM, Galberg P, Petersen K, O'shea C, Skriver K (2010). The Arabidopsis thaliana NAC transcription factor family: structure–function relationships and determinants of ANAC019 stress signalling. Biochem J.

[10] Olsen AN, Ernst HA, Leggio LL, Skriver K (2005). NAC transcription factors: structurally distinct, functionally diverse. Trends Plant Sci.

[11] Mathew IE, Agarwal P (2018). May the fittest protein evolve: favoring the plant-specific origin and expansion of NAC transcription factors. BioEssays.

[12] Christianson JA, Dennis ES, Llewellyn DJ, Wilson IW (2010). ATAF NAC transcription factors: regulators of plant stress signaling. Plant Signal Behav.

[13] Ooka H, Satoh K, Doi K, Nagata T, Otomo Y, Murakami K, Matsubara K, Osato N, Kawai J, Carninci P (2003). Comprehensive analysis of NAC family genes in *Oryza sativa* and *Arabidopsis thaliana*. DNA Res.

[14] Zhu G, Chen G, Zhu J, Zhu Y, Lu X, Li X, Hu Y, Yan Y (2015). Molecular characterization and expression profiling of NAC transcription factors in *Brachypodium distachyon* L. PLoS One..

[15] Zhu T, Nevo E, Sun D, Peng J (2012). Phylogenetic analyses unravel the evolutionary history of NAC proteins in plants. Evolution.

[16] Jensen MK, Skriver K (2014). NAC transcription factor gene regulatory and protein–protein interaction networks in plant stress responses and senescence. IUBMB Life.

[17] Guo H-S, Xie Q, Fei J-F, Chua N-H (2005). MicroRNA directs mRNA cleavage of the transcription factor NAC1 to downregulate auxin signals for Arabidopsis lateral root development. Plant Cell.

[18] Guo Y, Gan S (2006). AtNAP, a NAC family transcription factor, has an important role in leaf senescence. Plant J.

[19] Sablowski RW, Meyerowitz EM (1998). A homolog of NO APICAL MERISTEM is an immediate target of the floral homeotic genes APETALA3/PISTILLATA. Cell.

[20] Kim SG, Lee AK, Yoon HK, Park CM (2008). A membrane-bound NAC transcription factor NTL8 regulates gibberellic acid-mediated salt signaling in Arabidopsis seed germination. Plant J.

[21] Tian H, Wang X, Guo H, Cheng Y, Hou C, Chen J-G, Wang S (2017). NTL8 regulates trichome formation in Arabidopsis by directly activating R3 MYB genes TRY and TCL1. Plant Physiol.

[22] Kim JH, Woo HR, Kim J, Lim PO, Lee IC, Choi SH, Hwang D, Nam HG (2009). Trifurcate feed-forward regulation of age-dependent cell death involving miR164 in *Arabidopsis*. Science.

[23] Mathew IE, Das S, Mahto A, Agarwal P (2016). Three rice NAC transcription factors heteromerize and are associated with seed size. Front Plant Sci.

[24] Zhang J, Huang GQ, Zou D, Yan JQ, Li Y, Hu S, Li XB (2018). The cotton (*Gossypium hirsutum*) NAC transcription factor (FSN1) as a positive regulator participates in controlling secondary cell wall biosynthesis and modification of fibers. New Phytol.

[25] Zhong R, Richardson EA, Ye Z-H (2007). Two NAC domain transcription factors, SND1 and NST1, function redundantly in regulation of secondary wall synthesis in fibers of *Arabidopsis*. Planta.

[26] Mitsuda N, Iwase A, Yamamoto H, Yoshida M, Seki M, Shinozaki K, Ohme-Takagi M (2007). NAC transcription factors, NST1 and NST3, are key regulators of the formation of secondary walls in woody tissues of *Arabidopsis*. Plant Cell.

[27] Mitsuda N, Ohme-Takagi M (2008). NAC transcription factors NST1 and NST3 regulate pod shattering in a partially redundant manner by promoting secondary wall formation after the establishment of tissue identity. Plant J.

[28] Zhao Q, Gallego-Giraldo L, Wang H, Zeng Y, Ding SY, Chen F, Dixon RA (2010). An NAC transcription factor orchestrates multiple features of cell wall development in *Medicago truncatula*. Plant J.

[29] Seok H-Y, Woo D-H, Nguyen LV, Tran HT, Tarte VN, Mehdi SMM, Lee S-Y, Moon Y-H (2017). Arabidopsis AtNAP functions as a negative regulator via repression of AREB1 in salt stress response. Planta.

[30] He L, Shi X, Wang Y, Guo Y, Yang K, Wang Y (2017). Arabidopsis ANAC069 binds to C [a/G] CG [T/G] sequences to negatively regulate salt and osmotic stress tolerance. Plant Mol Biol.

[31] Chen D, Chai S, McIntyre CL, Xue G-P (2018). Overexpression of a predominantly root-expressed NAC transcription factor in wheat roots enhances root length, biomass and drought tolerance. Plant Cell Rep.

[32] Xue G-P, Way HM, Richardson T, Drenth J, Joyce PA, McIntyre CL (2011). Overexpression of TaNAC69 leads to enhanced transcript levels of stress up-regulated genes and dehydration tolerance in bread wheat. Mol Plant.

[33] Xu Z, Wang C, Xue F, Zhang H, Ji W (2015). Wheat NAC transcription factor TaNAC29 is involved in response to salt stress. Plant Physiol Biochem.

[34] Nakashima K, Tran LSP, Van Nguyen D, Fujita M, Maruyama K, Todaka D, Ito Y, Hayashi N, Shinozaki K, Yamaguchi-Shinozaki K (2007). Functional analysis of a NAC-type transcription factor OsNAC6 involved in abiotic and biotic stress-responsive gene expression in rice. Plant J.

[35] Hu H, You J, Fang Y, Zhu X, Qi Z, Xiong L (2008). Characterization of transcription factor gene SNAC2 conferring cold and salt tolerance in rice. Plant Mol Biol.

[36] Hong Y, Zhang H, Huang L, Li D, Song F (2016). Overexpression of a stress-responsive NAC transcription factor gene ONAC022 improves drought and salt tolerance in rice. Front Plant Sci.

[37] Thirumalaikumar VP, Devkar V, Mehterov N, Ali S, Ozgur R, Turkan I, Mueller-Roeber B, Balazadeh S (2018). NAC transcription factor JUNGBRUNNEN 1 enhances drought tolerance in tomato. Plant Biotechnol J.

[38] Yang X, He K, Chi X, Chai G, Wang Y, Jia C, Zhang H, Zhou G, Hu R (2018). Miscanthus NAC transcription factor MlNAC12 positively mediates abiotic stress tolerance in transgenic Arabidopsis. Plant Sci.

[39] Feng G, Huang L, Li J, Wang J, Xu L, Pan L, Zhao X, Wang X, Huang T, Zhang X (2017). Comprehensive transcriptome analysis reveals distinct regulatory programs during vernalization and floral bud development of orchardgrass (*Dactylis glomerata* L.). BMC Plant Biol.

[40] Huang L, Yan H, Jiang X, Zhang Y, Zhang X, Ji Y, Zeng B, Xu B, Yin G, Lee S (2014). Reference gene selection for quantitative real-time reverse-transcriptase PCR in orchardgrass subjected to various abiotic stresses. Gene.

[41] Casler M, Fales S, Undersander D, McElroy A (2001). Genetic progress from 40 years of orchardgrass breeding in North America measured under management-intensive rotational grazing. Can J Plant Sci.

[42] Shiriga K, Sharma R, Kumar K, Yadav SK, Hossain F, Thirunavukkarasu N (2014). Genome-wide identification and expression pattern of drought-responsive members of the NAC family in maize. Meta Gene.

[43] Le DT, Nishiyama R, Watanabe Y, Mochida K, Yamaguchi-Shinozaki K, Shinozaki K, Tran L-SP (2011). Genome-wide survey and expression analysis of the plant-specific NAC transcription factor family in soybean during development and dehydration stress. DNA Res.

[44] Singh AK, Sharma V, Pal AK, Acharya V, Ahuja PS (2013). Genome-wide organization and expression profiling of the NAC transcription factor family in potato (*Solanum tuberosum* L.). DNA Res.

[45] Gong X, Zhao L, Song X, Lin Z, Gu B, Yan J, Zhang S, Tao S, Huang X (2019). Genome-wide analyses and expression patterns under abiotic stress of NAC transcription factors in white pear (*Pyrus bretschneideri*). BMC Plant Biol.

[46] Liu M, Ma Z, Sun W, Huang L, Wu Q, Tang Z, Bu T, Li C, Chen H (2019). Genome-wide analysis of the NAC transcription factor family in Tartary buckwheat (*Fagopyrum tataricum*). BMC Genomics.

[47] Shan Z, Jiang Y, Li H, Guo J, Dong M, Zhang J, Liu G (2020). Genome-wide analysis of the NAC transcription factor family in broomcorn millet (*Panicum miliaceum* L.) and expression analysis under drought stress. BMC Genomics.

[48] Huang L, Feng G, Yan H, Zhang Z, Bushman BS, Wang J, Bombarely A, Li M, Yang Z, Nie G (2020). Genome assembly provides insights into the genome evolution and flowering regulation of orchardgrass. Plant Biotechnol J.

[49] Dalman K, Wind JJ, Nemesio-Gorriz M, Hammerbacher A, Lundén K, Ezcurra I, Elfstrand M (2017). Overexpression of PaNAC03, a stress induced NAC gene family transcription factor in Norway spruce leads to reduced flavonol biosynthesis and aberrant embryo development. BMC Plant Biol.

[50] Wang Z, Rashotte AM, Moss AG, Dane F (2014). Two NAC transcription factors from Citrullus colocynthis, CcNAC1, CcNAC2 implicated in multiple stress responses. Acta Physiol Plant.

[51] Wang Z, Rashotte AM, Dane F (2014). Citrullus colocynthis NAC transcription factors CcNAC1 and CcNAC2 are involved in light and auxin signaling. Plant Cell Rep.

[52] Diao W, Snyder JC, Wang S, Liu J, Pan B, Guo G, Ge W, Dawood MHSA (2018). Genome-wide analyses of the NAC transcription factor gene family in pepper (*Capsicum annuum* L.): chromosome location, phylogeny, structure, expression patterns, cis-elements in the promoter, and interaction network. Int J Mol Sci.

[53] Wei S, Gao L, Zhang Y, Zhang F, Yang X, Huang D (2016). Genome-wide investigation of the NAC transcription factor family in melon (*Cucumis melo* L.) and their expression analysis under salt stress. Plant Cell Rep.

[54] Hu W, Wei Y, Xia Z, Yan Y, Hou X, Zou M, Lu C, Wang W, Peng M (2015). Genome-wide identification and expression analysis of the NAC transcription factor family in cassava. PLoS One..

[55] Nuruzzaman M, Manimekalai R, Sharoni AM, Satoh K, Kondoh H, Ooka H, Kikuchi S (2010). Genome-wide analysis of NAC transcription factor family in rice. Gene.

[56] Liu T, Song X, Duan W, Huang Z, Liu G, Li Y, Hou X (2014). Genome-wide analysis and expression patterns of NAC transcription factor family under different developmental stages and abiotic stresses in Chinese cabbage. Plant Mol Biol Report.

[57] Blanc G, Hokamp K, Wolfe KH (2003). A recent polyploidy superimposed on older large-scale duplications in the Arabidopsis genome. Genome Res.

[58] Zhou Q, Zhang S, Chen F, Liu B, Wu L, Li F, Zhang J, Bao M, Liu G (2018). Genome-wide identification and characterization of the SBP-box gene family in *Petunia*. BMC Genomics.

[59] Naya FJ, Stellrecht C, Tsai M-J (1995). Tissue-specific regulation of the insulin gene by a novel basic helix-loop-helix transcription factor. Genes Dev.

[60] Clevidence DE, Overdier DG, Tao W, Qian X, Pani L, Lai E, Costa RH (1993). Identification of nine tissue-specific transcription factors of the hepatocyte nuclear factor 3/forkhead DNA-binding-domain family. Proc Natl Acad Sci.

[61] Ingraham HA, Chen R, Mangalam HJ, Elsholtz HP, Flynn SE, Lin CR, Simmons DM, Swanson L, Rosenfeld MG (1988). A tissue-specific transcription factor containing a homeodomain specifies a pituitary phenotype. Cell.

[62] Guerin C, Roche J, Allard V, Ravel C, Mouzeyar S, Bouzidi MF. Genome-wide analysis, expansion and expression of the NAC family under drought and heat stresses in bread wheat (*T. aestivum* L.). PLoS One. 2019;14(3):e0213390. 10.1371/journal.pone.0213390 PMC6402696 30840709

[63] Yao W, Zhang D, Zhou B, Wang J, Li R, Jiang T (2020). Over-expression of poplar NAC15 gene enhances wood formation in transgenic tobacco. BMC Plant Biol.

[64] Yang Y, Yoo CG, Rottmann W, Winkeler KA, Collins CM, Gunter LE, Jawdy SS, Yang X, Pu Y, Ragauskas AJ (2019). PdWND3A, a wood-associated NAC domain-containing protein, affects lignin biosynthesis and composition in *Populus*. BMC Plant Biol.

[65] Fan K, Wang M, Miao Y, Ni M, Bibi N, Yuan S, Li F, Wang X (2014). Molecular evolution and expansion analysis of the NAC transcription factor in *Zea mays*. PLoS One..

[66] Adams KL (2007). Evolution of duplicate gene expression in polyploid and hybrid plants. J Hered.

[67] Takada S, Hibara K-I, Ishida T, Tasaka M (2001). The CUP-SHAPED COTYLEDON1 gene of Arabidopsis regulates shoot apical meristem formation. Development.

[68] Kim Y-S, Kim S-G, Park J-E, Park H-Y, Lim M-H, Chua N-H, Park C-M (2006). A membrane-bound NAC transcription factor regulates cell division in Arabidopsis. Plant Cell.

[69] Duval M, Hsieh T-F, Kim SY, Thomas TL (2002). Molecular characterization of AtNAM: a member of theArabidopsis NAC domain superfamily. Plant Mol Biol.

[70] Nikovics K, Blein T, Peaucelle A, Ishida T, Morin H, Aida M, Laufs P (2006). The balance between the MIR164A and CUC2 genes controls leaf margin serration in Arabidopsis. Plant Cell.

[71] K-i H, Karim MR, Takada S, K-i T, Furutani M, Aida M, Tasaka M (2006). Arabidopsis CUP-SHAPED COTYLEDON3 regulates postembryonic shoot meristem and organ boundary formation. Plant Cell.

[72] Burian A, Raczyńska-Szajgin M, Borowska-Wykręt D, Piatek A, Aida M, Kwiatkowska D (2015). The CUP-SHAPED COTYLEDON2 and 3 genes have a post-meristematic effect on Arabidopsis thaliana phyllotaxis. Ann Bot.

[73] Ying L, Chen H, Cai W (2014). BnNAC485 is involved in abiotic stress responses and flowering time in *Brassica napus*. Plant Physiol Biochem..

[74] Ning Y-Q, Ma Z-Y, Huang H-W, Mo H, Zhao T-T, Li L, Cai T, Chen S, Ma L, He X-J (2015). Two novel NAC transcription factors regulate gene expression and flowering time by associating with the histone demethylase JMJ14. Nucleic Acids Res.

[75] Finn RD, Bateman A, Clements J, Coggill P, Eberhardt RY, Eddy SR, Heger A, Hetherington K, Holm L, Mistry J (2014). Pfam: the protein families database. Nucleic Acids Res.

[76] Finn RD, Clements J, Eddy SR (2011). HMMER web server: interactive sequence similarity searching. Nucleic Acid Res.

[77] Rhee SY, Beavis W, Berardini TZ, Chen G, Dixon D, Doyle A, Garcia-Hernandez M, Huala E, Lander G, Montoya M (2003). The Arabidopsis information resource (TAIR): a model organism database providing a centralized, curated gateway to Arabidopsis biology, research materials and community. Nucleic Acids Res.

[78] Guo A, Zhu Q, Chen X, Luo J (2007). GSDS: a gene structure display server. *Yi chuan=*. Hereditas.

[79] Bailey TL, Boden M, Buske FA, Frith M, Grant CE, Clementi L, Ren J, Li WW, Noble WS (2009). MEME SUITE: tools for motif discovery and searching. Nucleic Acid Res.

[80] Chen C, Chen H, Zhang Y, Thomas HR, Frank MH, He Y, Xia R (2020). TBtools: an integrative toolkit developed for interactive analyses of big biological data. Mol Plant.

[81] Reid KE, Olsson N, Schlosser J, Peng F, Lund ST (2006). An optimized grapevine RNA isolation procedure and statistical determination of reference genes for real-time RT-PCR during berry development. BMC Plant Biol.

[82] Zeng B, Zhang Y, Zhang A, Qiao D, Ren J, Li M, Cai K, Zhang J, Huang L (2020). Transcriptome profiling of two *Dactylis glomerata* L. cultivars with different tolerance in response to submergence stress. Phytochemistry.

[83] Ji Y, Chen P, Chen J, Pennerman KK, Liang X, Yan H, Zhou S, Feng G, Wang C, Yin G (2018). Combinations of small RNA, RNA, and Degradome sequencing uncovers the expression pattern of microRNA–mRNA pairs adapting to drought stress in leaf and root of *Dactylis glomerata *L. Int J Mol Sci.

[84] Huang L, Yan H, Zhao X, Zhang X, Wang J, Frazier T, Yin G, Huang X, Yan D, Zang W (2015). Identifying differentially expressed genes under heat stress and developing molecular markers in orchardgrass (*Dactylis glomerata* L.) through transcriptome analysis. Mol Ecol Resour.

[85] Kawahara Y, de la Bastide M, Hamilton JP, Kanamori H, McCombie WR, Ouyang S, Schwartz DC, Tanaka T, Wu J, Zhou S (2013). Improvement of the *Oryza sativa* Nipponbare reference genome using next generation sequence and optical map data. Rice..

[86] Jayakodi M, Padmarasu S, Haberer G, Bonthala VS, Gundlach H, Monat C, Lux T, Kamal N, Lang D, Himmelbach A, Ens J, Zhang XQ, Angessa TT, Zhou G, Tan C, Hill C, Wang P, Schreiber M, Boston LB, Plott C, Jenkins J, Guo Y, Fiebig A, Budak H, Xu D, Zhang J, Wang C, Grimwood J, Schmutz J, Guo G, Zhang G, Mochida K, Hirayama T, Sato K, Chalmers KJ, Langridge P, Waugh R, Pozniak CJ, Scholz U, KFX M, Spannagl M, Li C, Mascher M, Stein N (2020). The barley pan-genome reveals the hidden legacy of mutation breeding. Nature.

